# The Atlanta Society of Medicine

**Published:** 1890-10

**Authors:** 


### THE ATLANTA SOCIETY OF MEDICINE.

Our Society resumed its regular meetings on Tuesday evening, September 2, in the old hall. The President, Dr. Nicolson, presented his report upon the fulfilment of his promise to get a new hall by the fall meetings. On the evening of the 16th inst., the Society met in the new hall. The transformation of quarters was altogether pleasing. The hall is about four times as large as the old, and the furnishing such that the Society need in no wise feel ashamed to invite visitors. The membership is increasing, and renewed interest is markedly evident. Subjects brought up are well discussed, and there is no evidence of lukewarmness in the meetings.

The President has revived an old feature of the Society. At the last meeting he announced a subject for discussion at the second meeting following, and will appoint, in the meanwhile, leaders in the discussion; these to be followed by whosoever wishes to participate. This must prove of great interest to the members, and the taking up of each important subject during the winter will be highly instructive to all.

Prophecy regarding the future of our Society can only be favorable. The interest of each member, from the leading to the humblest, is all that is needed to raise the Society (figuratively) as far above its old self as the present hall is above our former humble quarters.

---

Dr. E. J. Roach, of this city, died September 11th, after a very short illness, at the age of fifty-eight years. He was a prominent member of various organizations, and was connected with the public school system, besides being engaged in the practice of his profession.

